# Exploring the Impact of miR-10a, miR-141, miR-200a and miR-3658 on Cystinuria

**DOI:** 10.3390/biomedicines14081688

**Published:** 2026-07-28

**Authors:** Daniel Cernach Ayres, Ruan Pimenta, Gabriel Arantes dos Santos, Patrícia Candido, Milena Antunes, Nelson Gaspar Dip Junior, Giovanni S. Marchini, Fábio C. Torricelli, Fábio C. Vicentini, Alexandre Danilovic, Carlos A. Batagello, Kátia R. M. Leite, William C. Nahas, Sabrina T. Reis, Eduardo Mazzucchi

**Affiliations:** 1 Laboratorio de Investigação Médica 55 (LIM55), Hospital das Clinicas, Faculdade de Medicina, Universidade de São Paulo (HCFMUSP), São Paulo 01246-903, SP, Brazil; 2 Precision Immunology Institute, Department of Immunology and Immunotherapy, and Tisch Cancer Institute, Icahn School of Medicine at Mount Sinai, New York, NY 10029, USA; 3 Moriah Institute of Science and Education (MISE), São Paulo 04084-002, SP, Brazil; 4 Endourology and Stone Disease Section, Division of Urology, Hospital das Clinicas, Faculdade de Medicina, Universidade de São Paulo (HCFMUSP), São Paulo 01246-903, SP, Brazil

**Keywords:** cystinuria, microRNA, nephrolithiasis, biomarkers, renal function

## Abstract

**Introduction**: Cystinuria is an inherited disorder characterized by impaired proximal tubular reabsorption of cystine, resulting in recurrent cystine stone formation. Mutations in the *SLC3A1* and *SLC7A9* genes are the main genetic causes of the disease, while variants in *PBX1* have also been associated with cystinuria in specific populations. Despite advances in understanding its molecular basis, cystinuria remains clinically heterogeneous, making disease progression and prognosis difficult to predict. This study aimed to investigate circulating microRNAs (miRNAs) as potential molecular biomarkers, characterize their expression profile in patients with cystinuria, and evaluate their association with clinical indicators of disease severity. **Methods**: The study group included 16 patients with a clinically established diagnosis of cystinuria, while the control group consisted of 11 healthy individuals without a history of nephrolithiasis. Candidate miRNAs were selected based on their predicted regulatory interactions with genes implicated in cystinuria pathogenesis. Eight microRNAs (miR-29a-3p, miR-29b-3p, miR-29c-3p, miR-1207-3p, miR-10a-3p, miR-3658, miR-141-3p, and miR-200a-3p) were analyzed by quantitative real-time PCR (qPCR). **Results:** Among the eight miRNAs evaluated, miR-10a, miR-141, miR-200a, and miR-3658 were significantly downregulated in patients with cystinuria compared with healthy controls. Higher expression levels of miR-10a, miR-141, and miR-200a were significantly associated with a greater number of previous surgical procedures (*p* < 0.05). Additionally, increased miR-10a expression was associated with impaired renal function, defined as serum creatinine levels above 1.2 mg/dL. **Conclusions:** miR-10a, miR-141, miR-200a, and miR-3658 were significantly downregulated in patients with cystinuria compared with healthy controls. Within the cystinuria cohort, higher expression of miR-10a was associated with impaired renal function, whereas increased expression of miR-10a, miR-141, and miR-200a correlated with a greater number of surgical interventions, suggesting a potential relationship with disease severity. Further studies in larger, genetically characterized cohorts are warranted to clarify the biological role and clinical utility of these miRNAs as biomarkers of cystinuria.

## 1. Introduction

Cystine stones account for approximately 1–2% of all urinary stone disease in adults but represent one of the most common inherited causes of nephrolithiasis, with a disproportionately high burden of recurrent stone formation and chronic kidney disease [1,2,3]. Although cystine calculi account for only approximately 1–2% of all urinary stones, cystinuria represents one of the most common inherited causes of nephrolithiasis and is associated with considerable lifelong morbidity owing to frequent stone recurrence, repeated surgical interventions, and progressive deterioration of renal function in a substantial proportion of affected patients [1,4].

The disease is caused predominantly by pathogenic variants in *SLC3A1* and *SLC7A9*, which encode the heavy (rBAT) and light subunits of the proximal tubular amino acid transporter responsible for cystine reabsorption [2,3]. More recently, additional genetic modifiers, including variants involving *PBX1*, have been proposed to contribute to disease susceptibility and phenotypic variability in selected populations [5]. Nevertheless, considerable clinical heterogeneity persists even among patients carrying similar pathogenic variants, indicating that mechanisms beyond the primary genetic defect probably influence disease expression and progression [6].

Current diagnosis relies on clinical history, quantitative urinary cystine excretion, stone composition analysis, urinary microscopy, and molecular testing when available [6,7]. Despite advances in genetic diagnosis, currently available biomarkers provide limited information regarding disease activity, recurrence risk, or long-term renal prognosis. Consequently, there remains an unmet need for molecular biomarkers capable of improving risk stratification and supporting individualized clinical management [6].

MicroRNAs (miRNAs) are endogenous non-coding RNAs that regulate gene expression at the post-transcriptional level through messenger RNA degradation or translational repression. Because individual miRNAs are capable of regulating multiple signaling pathways simultaneously, they play essential roles in inflammation, oxidative stress, fibrosis, epithelial homeostasis, cellular differentiation, and tissue remodeling [8,9,10]. In nephrology, numerous studies have demonstrated that circulating and urinary miRNAs represent promising biomarkers for chronic kidney disease, diabetic nephropathy, acute kidney injury, and renal fibrosis owing to their remarkable stability and close association with pathological processes [9,10].

Although the contribution of miRNAs has been extensively investigated in several renal disorders, their role in hereditary nephrolithiasis remains largely unexplored. To date, only limited evidence has evaluated miRNA expression in cystinuria, and no previous study has systematically investigated circulating miRNAs selected according to their predicted interactions with the principal genes implicated in cystinuria pathogenesis (*SLC3A1*, *SLC7A9*, and *PBX1*) [11]. Considering the growing recognition that epigenetic mechanisms may contribute to phenotypic variability in monogenic diseases, characterization of miRNA expression profiles may provide novel insights into disease biology while identifying potential biomarkers associated with disease severity [9].

Therefore, the present study aimed to evaluate the expression of circulating miRNAs predicted to regulate *SLC3A1*, *SLC7A9*, and *PBX1* in patients with clinically established cystinuria. Additionally, we investigated the association between miRNA expression and clinically relevant indicators of disease severity, including urinary cystine excretion, renal function, and the cumulative number of stone-related surgical procedures, to explore the potential biological and clinical significance of these molecules in cystinuria.

## 2. Methods

### 2.1. Patients and Ethics Statement

The patients were informed about the objectives of the study and the use of their biological samples for scientific research, and all participants provided written informed consent. The study protocol was approved by the Research Ethics Committee of the Faculty of Medicine, University of São Paulo (FMUSP) (approval number 3.524.405).

This study evaluated the expression profile of miRNAs in whole-blood samples obtained from 16 patients with clinically established cystinuria who were followed at the Endourology Outpatient Clinic of the Hospital das Clínicas, FMUSP. The diagnosis of cystinuria was established according to standard clinical criteria, including recurrent cystine stone disease, elevated 24 h urinary cystine excretion, characteristic stone composition when available, and evaluation by experienced endourologists. Although cystinuria is genetically heterogeneous, genetic testing was not routinely available for all patients because many had been diagnosed before molecular testing became incorporated into routine clinical practice. Therefore, patients were included based on a confirmed clinical diagnosis of cystinuria regardless of the underlying genotype.

The control group consisted of 11 healthy volunteers without a history of nephrolithiasis or chronic kidney disease. The demographic characteristics of the study population are presented in Table 1, whereas the detailed clinical characteristics of the cystinuria cohort are provided in Supplementary Table S1.

Clinical variables evaluated included 24 h urinary cystine excretion, serum creatinine levels, and the cumulative number of stone-related surgical procedures. Serum creatinine values corresponded to the laboratory assessment performed closest to blood collection. Patients were stratified according to the mean urinary cystine excretion of the study population (414 mg/day, rounded to 415 mg/day), the mean number of previous surgical procedures (12 procedures), and a serum creatinine cutoff of 1.2 mg/dL, corresponding to the upper limit of the normal reference range adopted by our institution. These thresholds were used exclusively for exploratory subgroup analyses of miRNA expression.

### 2.2. Selection of Candidate microRNAs

Candidate miRNAs were selected using a hypothesis-driven bioinformatic approach. TargetScan (https://www.targetscan.org) was used to identify miRNAs with predicted binding sites in the three genes investigated in this study (*SLC3A1*, *SLC7A9*, and *PBX1*). The miRNAs showing the strongest predicted interactions with each target gene were selected for subsequent expression analysis. Based on this strategy, three miRNAs targeting *SLC3A1* (hsa-miR-29a-3p, hsa-miR-29b-3p, and hsa-miR-29c-3p), three targeting *SLC7A9* (hsa-miR-1207-3p, hsa-miR-10a-3p, and hsa-miR-3658), and two targeting *PBX1* (hsa-miR-141-3p and hsa-miR-200a-3p) were included in the study.

### 2.3. MicroRNA Extraction and qPCR

Total RNA was extracted using the mirVana™ Kit (Ambion, Austin, TX, USA) according to the manufacturer’s instructions. RNA concentration and purity were assessed using a NanoDrop ND-1000 spectrophotometer (Thermo Fisher Scientific, Waltham, MA, USA). Complementary DNA (cDNA) was synthesized from the eight selected miRNAs using the TaqMan™ MicroRNA Reverse Transcription Kit (Applied Biosystems, Foster City, CA, USA), following the manufacturer’s recommendations. Quantitative real-time PCR (qPCR) was performed in a final reaction volume of 10 μL containing 2 μL of HOT FIREPol^®^ Probe Universal qPCR Mix (Solis BioDyne, Tartu, Estonia) and the corresponding TaqMan™ MicroRNA Assays (Applied Biosystems, Foster City, CA, USA) (Table 2). Thermal cycling conditions consisted of 2 min at 50 °C, 10 min at 95 °C, followed by amplification cycles according to the manufacturer’s protocol. RNU48 was used as the endogenous control for normalization. Relative miRNA expression was calculated using the 2^−ddct^ method. All reactions were performed in duplicate, and data were analyzed using DataAssist Software v3.01 (Applied Biosystems, Foster City, CA, USA).

### 2.4. Statistical Analysis

Statistical analyses were performed using GraphPad Prism version 9.0 (GraphPad Software, San Diego, CA, USA). Data normality was assessed using the Shapiro–Wilk test. Parametric variables were analyzed using the unpaired Student’s *t*-test, whereas non-parametric variables were compared using the Mann–Whitney *U* test. All statistical tests were two-tailed, and a *p* value < 0.05 was considered statistically significant. Because this was an exploratory study evaluating the expression of multiple candidate miRNAs, statistical analyses were interpreted as hypothesis-generating and should be considered exploratory in nature.

## 3. Results

### 3.1. Clinical–Demographic Characteristics

The clinical–demographic characteristics of the cystinuria patients included mean cystinuria dosage values in 24 h urine of 414 mg/24 h, with a median of 432 mg/24 h, ranging from 98 mg/24 h to 731 mg/24 h. The average number of surgical procedures that patients with cystinuria underwent was 12.06. The procedures performed were Extracorporeal Shock Wave Lithotripsy (ESWL) (34%), Percutaneous Nephrolithotripsy (PCNL) (29%), Ureterorenolithotripsy (URL) (26%) and open surgeries (11%). The mean creatinine value was 1.5 mg/dL, with a median of 1.005, ranging between 0.6 mg/dL and 5.11 mg/dL.

### 3.2. Expression Pattern of microRNAs in Cystinuria Patients

**Figure 1:**
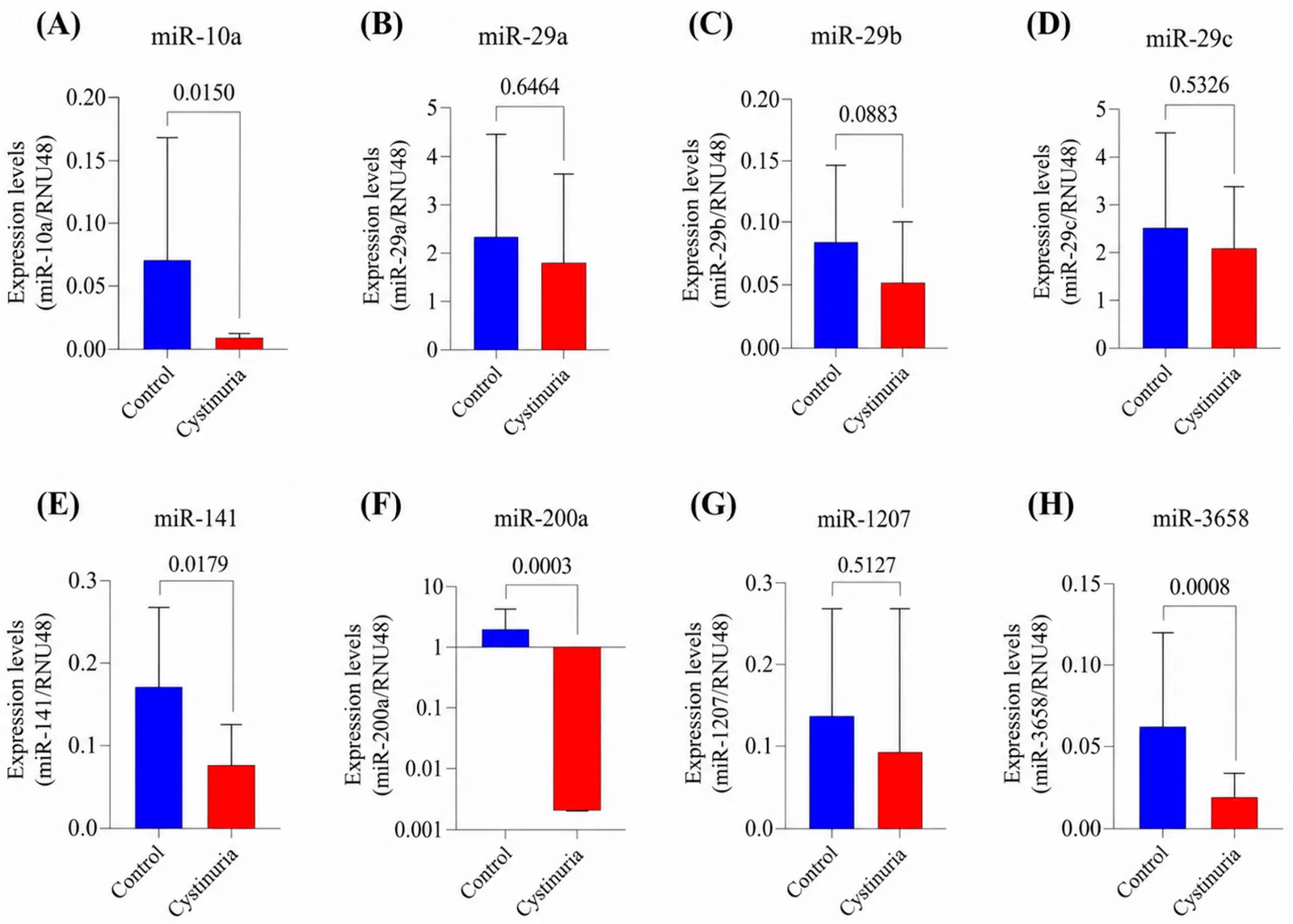
The relative expression profile of the evaluated miRNAs in patients with cystinuria and healthy controls. (**A**) miR-29a-3p; (**B**) miR-29b-3p; (**C**) miR-29c-3p; (**D**) miR-1207-3p; (**E**) miR-10a-3p; (**F**) miR-141-3p; (**G**) miR-200a-3p; and (**H**) miR-3658.

Figure 1 shows the expression profile of the eight candidate miRNAs in patients with cystinuria and healthy controls. Overall, all evaluated miRNAs exhibited lower expression in the cystinuria group, although statistically significant differences were observed only for miR-10a, miR-141, miR-200a, and miR-3658.

Compared with healthy controls, miR-10a expression was reduced approximately 14-fold in patients with cystinuria (*p* = 0.0150; Figure 1A). Similarly, miR-141 expression was decreased by approximately 18-fold (*p* = 0.0179; Figure 1F), whereas miR-200a showed the greatest reduction, with an approximately 1200-fold decrease (*p* = 0.0003; Figure 1G). In addition, miR-3658 expression was significantly reduced approximately 114-fold compared with controls (*p* = 0.0008; Figure 1H).

### 3.3. Expression Profile of microRNAs in Relation to Cystine Excretion in Patients with Cystinuria

**Figure 2:**
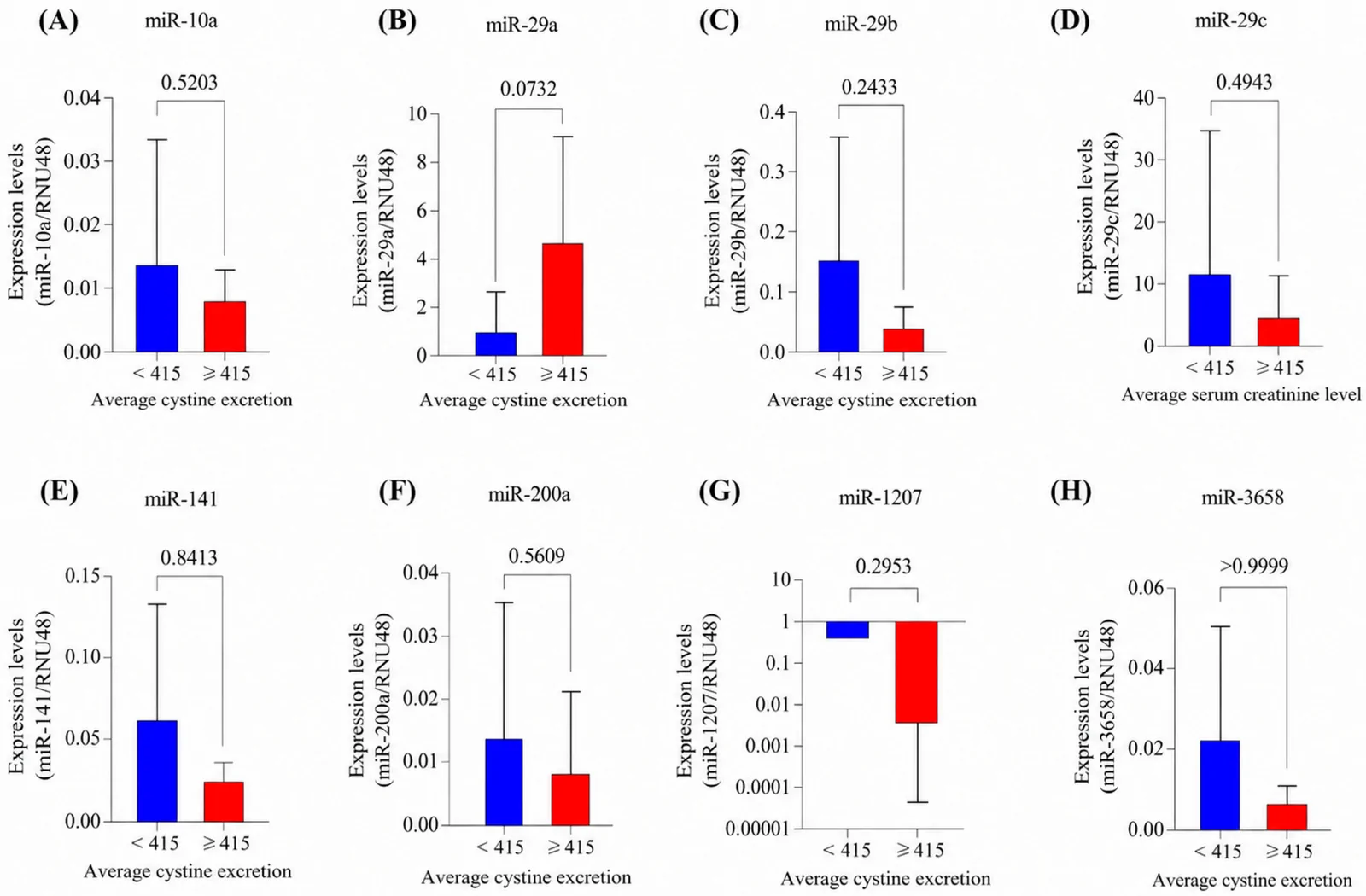
Association between miRNA expression levels and 24 h urinary cystine excretion in patients with cystinuria. (**A**) miR-29a-3p; (**B**) miR-29b-3p; (**C**) miR-29c-3p; (**D**) miR-1207-3p; (**E**) miR-10a-3p; (**F**) miR-141-3p; (**G**) miR-200a-3p; and (**H**) miR-3658.

To investigate whether miRNA expression was associated with disease biochemical severity, patients were stratified according to the mean 24 h urinary cystine excretion (415 mg/day). No statistically significant differences in the expression of any of the evaluated miRNAs were observed between patients with lower and higher urinary cystine excretion (Figure 2).

### 3.4. Expression of microRNAs in Relation to the Number of Surgical Procedures

**Figure 3:**
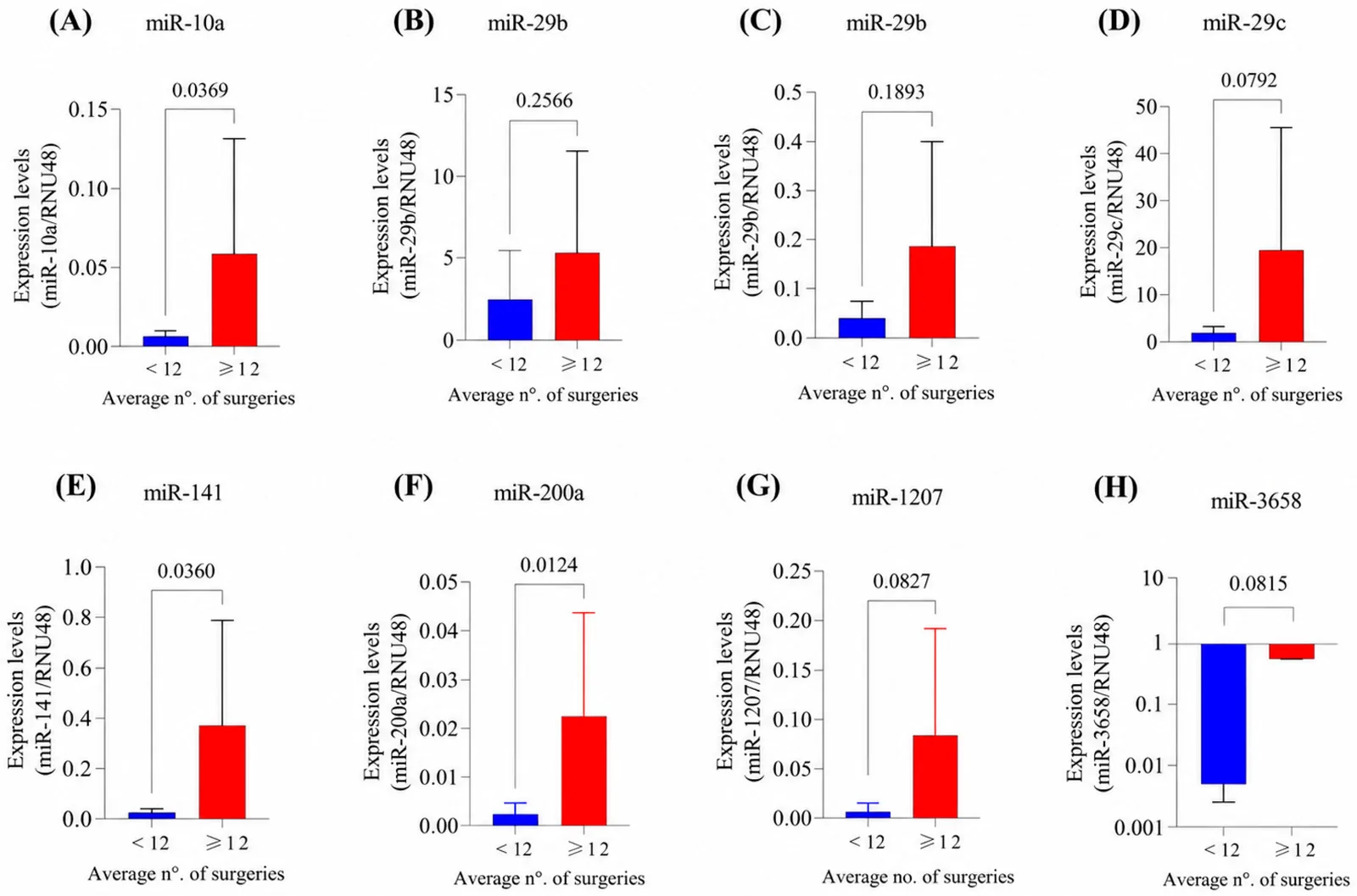
Association between miRNA expression levels and number of surgical procedures in patients with cystinuria. (**A**) miR-29a-3p; (**B**) miR-29b-3p; (**C**) miR-29c-3p; (**D**) miR-1207-3p; (**E**) miR-10a-3p; (**F**) miR-141-3p; (**G**) miR-200a-3p; and (**H**) miR-3658.

Patients were subsequently stratified according to the mean number of previous stone-related surgical procedures (12 procedures). As shown in Figure 3, higher expression of miR-10a was significantly associated with a greater number of previous surgical interventions (*p* = 0.0369; Figure 3A). Similarly, increased expression of miR-141 was observed in patients who had undergone a higher number of surgical procedures (*p* = 0.0360; Figure 3F). Likewise, miR-200a expression was significantly higher in patients with a greater surgical burden (*p* = 0.0124; Figure 3G).

### 3.5. Analysis of microRNA Expression and Its Association with Creatinine Excretion

**Figure 4:**
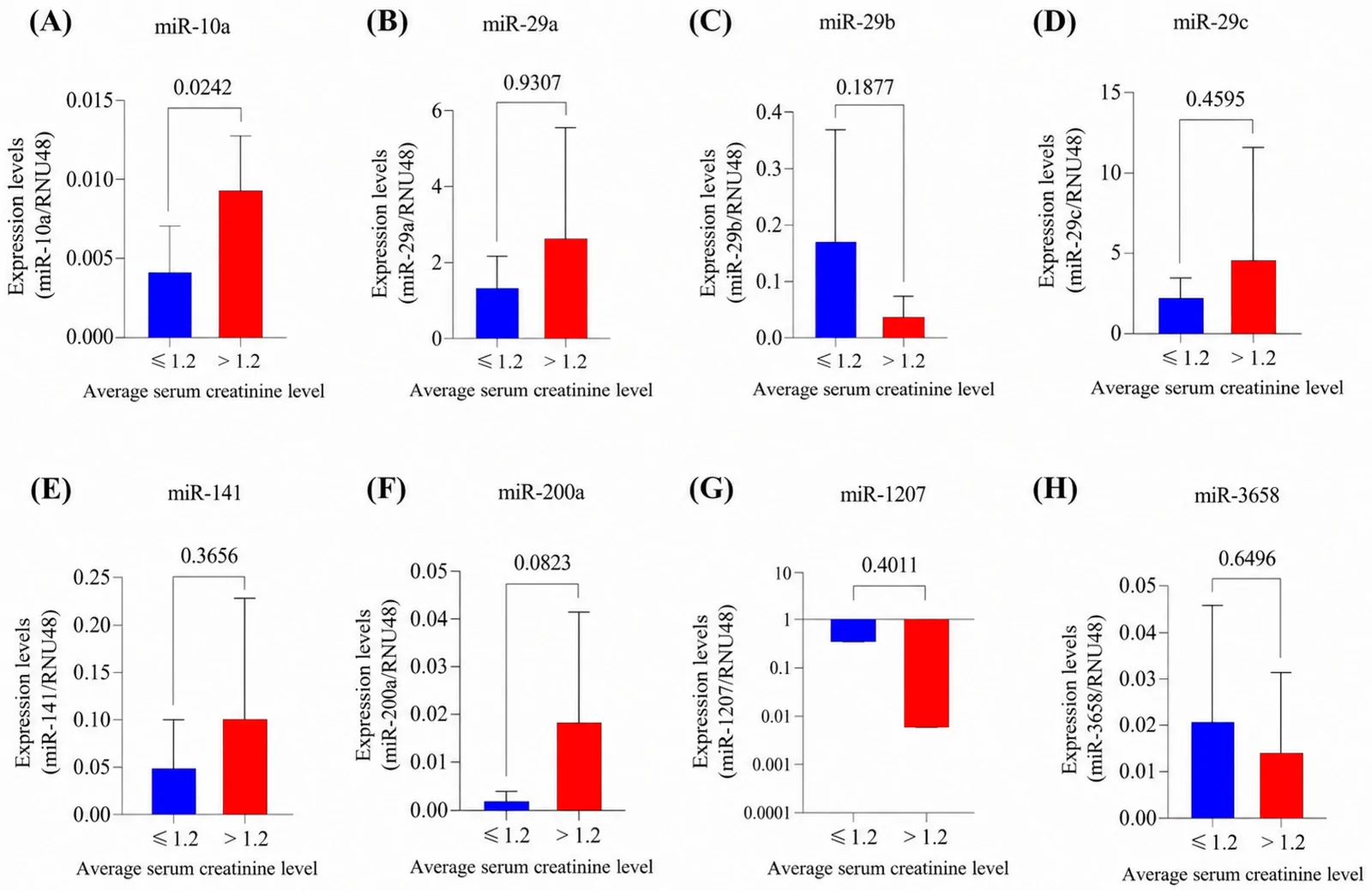
Association between miRNA expression levels and renal function in patients with cystinuria according to serum creatinine levels (mg/dL). (**A**) miR-29a-3p; (**B**) miR-29b-3p; (**C**) miR-29c-3p; (**D**) miR-1207-3p; (**E**) miR-10a-3p; (**F**) miR-141-3p; (**G**) miR-200a-3p; and (**H**) miR-3658.

To evaluate the relationship between miRNA expression and renal function, patients were categorized according to serum creatinine levels using a cutoff value of 1.2 mg/dL. Among all evaluated miRNAs, only miR-10a demonstrated a statistically significant association with renal function. Patients with serum creatinine levels above 1.2 mg/dL exhibited significantly higher miR-10a expression than those with preserved renal function (Figure 4A).

## 4. Discussion

Cystinuria is a rare inherited disorder primarily associated with mutations in the *SLC3A1* and *SLC7A9* genes, leading to recurrent cystine stone formation and progressive renal damage in a subset of patients. Although variants in *PBX1* have also been associated with susceptibility to cystinuria in specific populations [4], the disease remains genetically heterogeneous, and factors beyond the primary genetic defect are likely to contribute to its variable clinical presentation. Despite the well-established genetic basis of cystinuria, current diagnostic approaches remain largely dependent on clinical evaluation, stone analysis, urinary cystine quantification, and, when available, molecular testing [5]. In the present study, we evaluated the expression of miRNAs predicted to regulate genes involved in cystinuria pathogenesis, including *PBX1*, revealing significant downregulation of miR-10a, miR-141, miR-200a, and miR-3658 in patients with cystinuria. These findings suggest that epigenetic mechanisms may contribute to disease pathophysiology and reinforce the potential role of miRNAs as molecular biomarkers in this hereditary disorder.

The identification of reduced miR-10a expression in patients with cystinuria provides additional insight into the molecular mechanisms that may underlie disease progression. Previous studies have demonstrated that miR-10a plays an important role in renal diseases, particularly by modulating inflammatory and fibrotic pathways involved in kidney injury [10,11]. More specifically, miR-10a has been shown to regulate inflammatory signaling through inhibition of the NLRP3 inflammasome, thereby reducing cytokine production, inflammatory cell infiltration, and tissue damage [10,11]. Therefore, the downregulation observed in our cohort may contribute to persistent inflammatory activity associated with recurrent cystine stone formation and chronic renal injury. Interestingly, although miR-10a was globally downregulated in patients with cystinuria compared with healthy controls, patients with impaired renal function and a greater number of previous surgical procedures exhibited relatively higher miR-10a expression. This apparently paradoxical finding may reflect a compensatory response to chronic renal injury rather than a primary pathogenic mechanism. Similar dynamic changes in miRNA expression have been described during the progression of chronic kidney diseases, in which regulatory miRNAs may become partially upregulated during advanced stages of inflammation and tissue remodeling. Although this hypothesis requires experimental confirmation, it provides a plausible biological explanation for our findings and addresses the heterogeneous clinical behavior commonly observed in cystinuria.

The marked downregulation of miR-200a (approximately 1200-fold) and miR-141 (approximately 18-fold) observed in patients with cystinuria further supports the existence of an altered molecular profile associated with this disease. Previous studies by Xiong et al. demonstrated that members of the miR-200 family play an important role in preventing renal fibrosis by inhibiting transforming growth factor-β (TGF-β)-induced epithelial-to-mesenchymal transition through regulation of *ZEB1* and *ZEB2* expression [12]. Consequently, reduced expression of these miRNAs may increase susceptibility to chronic renal remodeling and fibrosis, both of which are recognized complications of recurrent cystine nephrolithiasis. However, similar to miR-10a, patients who had undergone a greater number of surgical procedures exhibited relatively higher expression of miR-141 and miR-200a. Rather than contradicting the overall downregulation observed in the cystinuria cohort, this finding may indicate that repeated renal injury, chronic inflammation, and tissue repair activate compensatory molecular responses involving these regulatory miRNAs. Because the present study has a cross-sectional design, it is not possible to determine whether these expression changes represent adaptive mechanisms or simply reflect disease severity. Longitudinal studies will be necessary to clarify these dynamic molecular alterations.

The significant downregulation of miR-3658 in patients with cystinuria compared with healthy controls raises important questions regarding its biological role in renal pathophysiology. Although miR-3658 has been extensively investigated in cancer biology, particularly in bladder cancer, where it has been associated with cell proliferation, migration, invasion, and reduced chemosensitivity [13], there is currently no evidence linking this miRNA to nephrolithiasis or cystinuria. To the best of our knowledge, this is the first study to demonstrate altered circulating miR-3658 expression in patients with cystinuria. Although its biological function in renal tissue remains unknown, the marked downregulation observed in our cohort suggests that this miRNA may participate in molecular pathways associated with chronic renal injury or stone disease. Further functional studies are necessary to determine whether miR-3658 directly regulates pathways involved in cystine stone formation or represents an epiphenomenon secondary to chronic renal damage.

The association between cystinuria and chronic kidney disease (CKD) highlights the long-term clinical burden of this hereditary disorder. Recurrent stone formation, repeated urinary tract obstruction, multiple surgical interventions, and recurrent urinary infections contribute to progressive deterioration of renal function in a considerable proportion of patients. In the present study, patients presented a mean serum creatinine level of 1.5 mg/dL (median 1.005 mg/dL; range 0.6–5.11 mg/dL), reflecting the heterogeneous degree of renal impairment observed within our cohort. Previous studies have demonstrated that several circulating miRNAs become dysregulated during CKD progression, suggesting that these molecules may serve as biomarkers of renal injury [13,14]. Therefore, the increased expression of miR-10a observed in patients with impaired renal function may reflect ongoing tissue remodeling and chronic inflammatory activity rather than being exclusively related to cystinuria itself. Because our control group consisted of healthy individuals without chronic kidney disease, we cannot completely exclude the possibility that part of the observed miRNA alterations may be attributable to renal dysfunction rather than cystinuria alone. This limitation should be considered when interpreting our findings and highlights the importance of future studies including disease-control groups with CKD unrelated to cystinuria.

When evaluating the relationship between miRNA expression and previous stone-related surgical procedures, we observed that patients with a greater surgical burden exhibited significantly higher expression of miR-10a, miR-141, and miR-200a. The cutoff value used for subgroup analysis corresponded to the mean number of surgical procedures performed in our cohort. Because recurrent surgical intervention reflects cumulative disease burden rather than isolated stone episodes, these findings suggest that the expression of these miRNAs may be associated with disease progression. Previous studies have demonstrated that miR-141 and miR-200a participate in pathways regulating renal fibrosis and extracellular matrix remodeling [13,15,16]. Therefore, the relatively higher expression observed in patients with more extensive surgical histories may represent an adaptive response to repetitive renal injury and fibrosis rather than a direct consequence of stone formation itself. This hypothesis is consistent with the dynamic behavior of regulatory miRNAs described in several chronic kidney diseases, in which expression patterns vary according to disease stage and the balance between tissue injury and repair [15,16].

Another important aspect that should be considered is the genetic heterogeneity of cystinuria. Because comprehensive molecular characterization was not available for all patients included in this study, we were unable to investigate whether different genetic backgrounds influence miRNA expression profiles. Likewise, the present study focused on circulating miRNAs predicted to regulate *SLC3A1, SLC7A9*, and *PBX1* based on bioinformatic analysis and did not evaluate the expression of these target genes. Future studies combining genotyping, mRNA expression, and miRNA profiling in larger cohorts will be essential to better define the regulatory networks involved in cystinuria pathogenesis.

Despite the substantial impact of cystinuria on renal function and the high morbidity associated with recurrent stone formation, studies investigating the molecular mechanisms underlying this disease remain scarce. To date, few studies have explored the role of circulating miRNAs in cystinuria or examined their relationship with clinically relevant outcomes. Therefore, the present study expands the current understanding of the molecular landscape of cystinuria by demonstrating that miR-10a, miR-141, miR-200a, and miR-3658 are differentially expressed in affected patients and that some of these miRNAs are associated with markers of disease severity, including renal function and cumulative surgical burden. These findings suggest that circulating miRNAs may represent promising biomarkers for patient stratification and prognosis.

Nevertheless, the present results should be interpreted considering several limitations. First, this was an exploratory study performed in a relatively small cohort of patients with a rare disease. Second, because the control group consisted exclusively of healthy individuals without nephrolithiasis or chronic kidney disease, it was not possible to determine whether the observed miRNA alterations are entirely specific to cystinuria or partially reflect chronic renal dysfunction. Third, genetic characterization was not available for all patients, precluding subgroup analyses according to the underlying molecular defect. Finally, although the investigated miRNAs were selected based on bioinformatic prediction of interactions with genes involved in cystinuria pathogenesis, the expression of the target genes (*SLC3A1, SLC7A9*, and *PBX1*) was not evaluated in the present study. Future investigations integrating genomic, transcriptomic, and miRNA analyses in larger multicenter cohorts will be essential to clarify the biological mechanisms underlying these associations.

Although the clinical application of circulating miRNAs remains challenging, their potential as minimally invasive biomarkers is increasingly recognized in several renal diseases. Unlike conventional biochemical markers, miRNAs may provide additional information regarding disease activity, inflammatory status, and tissue remodeling before irreversible renal damage becomes clinically evident. If validated in independent cohorts, the miRNAs identified in the present study may contribute to improved risk stratification of patients with cystinuria, allowing for closer surveillance of individuals at greater risk of renal function decline and recurrent surgical intervention. Furthermore, advances in miRNA-based therapeutics may eventually provide novel opportunities for targeted molecular interventions aimed at modulating inflammatory and fibrotic pathways involved in cystinuria progression. However, additional mechanistic and longitudinal studies are necessary before these molecules can be translated into routine clinical practice.

## 5. Conclusions

The present study demonstrated that circulating miR-10a, miR-141, miR-200a, and miR-3658 are significantly downregulated in patients with cystinuria compared with healthy controls. In addition, higher expression of miR-10a was associated with impaired renal function, whereas increased expression of miR-10a, miR-141, and miR-200a was associated with a greater number of previous stone-related surgical procedures, suggesting a relationship with disease severity.

Although these findings require validation in larger and genetically characterized cohorts, they provide preliminary evidence that circulating miRNAs may represent promising biomarkers for the molecular characterization and prognostic assessment of patients with cystinuria. Future studies integrating miRNA expression with genomic and transcriptomic analyses may contribute to a better understanding of cystinuria pathogenesis and support the development of more personalized approaches for disease monitoring and management.

## Figures and Tables

**Table 1:** Demographic characteristics of study groups.

Cystinuria (*n* = 16)			Control (*n* = 11)	
Gender (%)	Male (50)	Female (50)	Male (36.3)	Female (63.7)
**Age**				
Mean−Median	35.5–27.5	45–44	36.3–38	43–40
Min−Max	16–67	26–70	27–42	22–62

**Table 2:** MicroRNAs analyzed in study, corresponding TaqMan™ assay IDs, and predicted target genes.

MicroRNA and Genes	TaqMan Assay	Target Gene
Hsa-miR 29a-3p	002112	*SLC3A1*
Hsa-miR 29b-3p	000413	*SLC3A1*
Hsa-miR 29c-3p	000415	*SLC3A1*
Hsa-miR 1207-3p	002826	*SLC7A9*
Hsa-miR 10a-3p	000387	*SLC7A9*
Hsa-miR 3658	4440886	*SLC7A9*
Hsa-miR 141-3p	000463	*PBX1*
Hsa-miR 200a-3p	000502	*PBX1*
RNU 48	1006	-

## Data Availability

The data presented in this study are available from the corresponding author on request.

## Supplementary Materials

The following supporting information can be downloaded at: https://www.mdpi.com/article/10.3390/biomedicines14081688/s1, Supplementary Table S1: Clinical characteristics of patients with cystinuria.
