# Supplementary material for: Exploring the Impact of miR-10a, miR-141, miR-200a and miR-3658 on Cystinuria
Source: Biomedicines. 2026 Jul 28;14(8):1688. doi: 10.3390/biomedicines14081688 (PMC13509378; doi:10.3390/biomedicines14081688)
Supplement: Supplementary file 1 [file biomedicines-14-01688-s001.zip › biomedicines-4404548-supplementary.pdf]

SUPPLEMENTAL MATERIAL

Supplementary Table S1. Characteristics of patients being monitored with cystinuria.

Patient	Sex	Age (Current/Diagnosis, years)	Initial Presentation	Family History	Solitary Kidney	24-h Urinary Cystine (mg/day)	Medical Treatment
Cis 1	F	61 / 28	Flank pain	No	No	NA	None
Cis 2	M	25 / 6	Hematuria	Yes	No	541	Potassium citrate
Cis 3	F	26 / 14	Flank pain	Yes	No	648	Potassium citrate
Cis 4	M	48 / 40	Flank pain	No	No	407	None
Cis 5	M	30 / 28	Flank pain	No	No	449	None
Cis 6	F	53 / 48	Flank pain	Yes	Yes	731	Potassium citrate + D-penicillamine
Cis 7	F	41 / 7	Flank pain	No	Yes	98	Potassium citrate
Cis 8	M	67 / 8	Flank pain	Yes	Yes	382	None
Cis 9	F	42 / 29	Flank pain	No	No	130	None
Cis 10	F	70 / 19	Flank pain	Yes	No	419	None
Cis 11	M	47 / 30	Flank pain	No	No	450	Sodium bicarbonate + Captopril
Cis 12	F	30 / 13	Flank pain	No	No	404	Potassium citrate
Cis 13	F	32 / 5	Flank pain	No	Yes	445	Potassium citrate + D-penicillamine
Cis 14	M	23 / 9	Flank pain	No	No	301	Potassium citrate + Captopril
Cis 15	M	16 / 16	Urinary retention	No	No	150	Potassium citrate + Captopril
Cis 16	M	53 / 30	Flank pain	Yes	Yes	NA	Potassium citrate + Captopril
